# Fat mass is associated with disabling foot pain

**DOI:** 10.1186/1757-1146-4-S1-P54

**Published:** 2011-05-20

**Authors:** Stephanie K Tanamas, Anita E Wluka, Patricia Berry, Hylton B Menz, Boyd J Strauss, Miranda Davies-Tuck, Joseph Proietto, John B Dixon, Graeme Jones, Flavia M Cicuttini

**Affiliations:** 1 School of Public Health and Preventive Medicine, Monash University, Alfred Hospital, Melbourne, Victoria 3004, Australia; 2 Musculoskeletal Research Centre, Faculty of Health Sciences, La Trobe University, Victoria 3086, Australia; 3 Departments of Medicine and Nutrition and Dietetics, Monash University, Southern Clinical School, Victoria 3168, Australia; 4 Department of Medicine, Repatriation Hospital, Heidelberg, Victoria 3081, Australia; 5 Baker IDI Heart and Diabetes Institute, Melbourne, Victoria 3004, Australia; 6 Menzies Research Institute, Private Bag 23, Hobart, Tasmania, Australia

## Background

Several studies have reported associations between increased body mass index (BMI) and foot pain. However, it is unclear as to whether the mechanism underlying this association is mechanical, metabolic, or both. Therefore, the aim of this study was to examine the relationship between body composition and disabling foot pain.

## Methods

137 subjects aged 25 to 62 years were recruited across a range of BMI (17 to 50 kg/m^2^) as part of a study examining the relationship between obesity and musculoskeletal health. Disabling foot pain was determined from the Manchester Foot Pain and Disability Index (MFPDI) and was defined as current foot pain and pain in the last month, as well as recording at least one disability item on the MFPDI. Body composition was measured using dual x-ray absorptiometry.

## Results

The BMI in this population was normally distributed around a mean of 32.2 kg/m^2^.The risk of disabling foot pain was associated with total fat mass (p<0.001) and skeletal muscle mass (p=0.01). However, when both total fat and skeletal muscle mass were included in the model, the relationship persisted for total fat mass but not muscle mass. The risk of disabling foot pain increased by 6% (95% confidence interval 2 - 9%) for every kilogram increase of total body fat, adjusted for age, gender, physical activity and skeletal muscle mass.

## Conclusions

Fat mass, rather than muscle mass, is associated with foot pain and disability. This suggests that the effect of obesity on foot pain may not only be due to increased loading on the foot, but may also act via a metabolic mechanism through increased adiposity. Further work is needed to clarify the mechanisms for this effect and the relative importance of reducing body fat rather than simple weight loss in the reduction of foot pain.

